# Nuclear Import and Export Signals of Human Cohesins SA1/STAG1 and SA2/STAG2 Expressed in *Saccharomyces cerevisiae*

**DOI:** 10.1371/journal.pone.0038740

**Published:** 2012-06-08

**Authors:** Leszek J. Tarnowski, Piotr Kowalec, Michał Milewski, Marta Jurek, Danuta Plochocka, Jan Fronk, Anna Kurlandzka

**Affiliations:** 1 Institute of Biochemistry and Biophysics, Polish Academy of Sciences, Warsaw, Poland; 2 Institute of Biochemistry, Faculty of Biology, University of Warsaw, Warsaw, Poland; 3 Department of Medical Genetics, Institute of Mother and Child, Warsaw, Poland; Russian Academy of Sciences, Institute for Biological Instrumentation, Russian Federation

## Abstract

**Background:**

Human SA/STAG proteins, homologues of the yeast Irr1/Scc3 cohesin, are the least studied constituents of the sister chromatid cohesion complex crucial for proper chromosome segregation. The two SA paralogues, SA1 and SA2, show some specificity towards the chromosome region they stabilize, and SA2, but not SA1, has been shown to participate in transcriptional regulation as well. The molecular basis of this functional divergence is unknown.

**Methodology/Principal Findings:**

*In silico* analysis indicates numerous putative nuclear localization (NLS) and export (NES) signals in the SA proteins, suggesting the possibility of their nucleocytoplasmic shuttling. We studied the functionality of those putative signals by expressing fluorescently tagged SA1 and SA2 in the yeast *Saccharomyces cerevisiae*. Only the N-terminal NLS turned out to be functional in SA1. In contrast, the SA2 protein has at least two functional NLS and also two functional NES. Depending on the balance between these opposing signals, SA2 resides in the nucleus or is distributed throughout the cell. Validation of the above conclusions in HeLa cells confirmed that the same N-terminal NLS of SA1 is functional in those cells. In contrast, in SA2 the principal NLS functioning in HeLa cells is different from that identified in yeast and is localized to the C-terminus.

**Conclusions/Significance:**

This is the first demonstration of the possibility of non-nuclear localization of an SA protein. The reported difference in the organization between the two SA homologues may also be relevant to their partially divergent functions. The mechanisms determining subcellular localization of cohesins are only partially conserved between yeast and human cells.

## Introduction

Division of the eukatryotic cell requires exact distribution of a proper number of chromosomes into both daughter cells during mitosis. This involves, among others, tight coupling of sister chromatids until the early or mid-phase of mitosis and then their concerted separation at the onset of anaphase. One of the principal mechanisms responsible for the association of chromatids prior to their segregation is sister chromatid cohesion that relies on a complex of proteins highly conserved from yeasts to mammals, called cohesin. This complex consists of four core subunits: the structural maintenance of chromosomes (SMC) proteins SMC1 (Smc1 in the yeast *Saccharomyces cerevisiae*) and SMC3 (Smc3), the kleisin SCC1/RAD21 (Scc1/Rad21/Mcd1), and the least studied HEAT-repeat domain subunit called Irr1/Scc3 in yeast and SA/STAG or stromalins in humans. The basic function of the SA proteins has been elucidated owing to findings on the role of their yeast orthologue Irr1p [1]. Human cells contain two mitotic equivalents of Irr1, SA1 and SA2 (also called STAG1 and STAG2), which are present in two distinct 14S cohesin complexes [2]–[4]. Initially it was shown that these two complexes differ by their SA constituent, SA1 or SA2, and it was assumed that only one type of SA is present in a given cell [2], [5], [6]. However, subsequent data obtained by Canudas and Smith [7] indicated that both SA1 and SA2 were in fact present in one cell and were specifically required for the cohesion of chromosome arms and telomeres, and centromeres, respectively. Recent stoichiometry data indicate that in HeLa cells the ratio of complexes containing SA1 and SA2 is in the range between 1∶12 and 1∶15 [8].

Several topological models of sister chromatid cohesion have been proposed (for review: [9]). The most popular one-ring model assumes that the complex surrounds the replicated sister chromatids, with SMC1 and SMC3 creating a V-shaped heterodimer bridged by SCC1 [10]; [11]. Another model posits that the complex consists of two cohesin rings, each encircling a single chromatid, that are paired through an interaction of the C-terminal domain of SCC1 with SA [6].

In mammalian cells most cohesin complexes are associated with chromatin. However, a large fraction of cohesin on chromosome arms dissociates already during prophase, while the cohesin at the centromeres remains bound until the metaphase-to-anaphase transition. Gerlich et al. [12] identified three sub-populations of cohesin complexes whose SA proteins differed in their dynamic equilibrium rate between chromatin-bound and soluble state. Also in yeast the core cohesin subunits are dynamic. They are able to bind to and dissociate from chromatin and, potentially, to dissociate/associate from the whole cohesin complex *in vivo*. These events take place on a time scale less than a cell cycle, but without the loss of chromosome cohesion [13]. Metazoan cells undergo open mitosis with the microtubule-organizing centers, centrosomes, located outside the nucleus. At the onset of mitosis (prometaphase), the nuclear envelope breaks down and nuclear pore complexes disassemble. This enables microtubules of the spindle to interact with centrosomes [14], [15]. Also at this stage of mitosis a large fraction of cohesin dissociates from chromosome arms.

In addition to its originally identified function in ensuring cohesion of chromatids, the SA2 protein takes part in regulating transcription, where it can function as a co-activator [16] as well as element of the insulator complex [17]. The molecular mechanisms enabling such diverse activities and the functional distinction between SA1 and SA2 are poorly understood.

We undertook to address these questions by using a simplified model of the yeast cell for initial experiments. Budding yeast, as many other single-cell *Eukaryota*, undergo closed mitosis throughout which their nuclear envelope remains intact [18]. Although closed mitosis is different from the open one in a number of features, the fundamental mechanisms of chromosome segregation are preserved, and the continuous presence of the nuclear envelope facilitates studies of a crucial aspect of the process, namely nucleo-cytoplasmic distribution of proteins. We took advantage of this aspect of yeast mitosis and also of the fact that it expresses only a single SA orthologue to study functional differences between the human SA paralogues. SA1 and SA2 were expressed individually in *S. cerevisiae* and their subcellular localization was investigated. Since our *in silico* analysis indicated that several nuclear localization signals (NLS) are present in SA1 and SA2, we investigated their functionality. Unexpectedly, also putative nuclear export signals (NES) were detected, suggesting the possibility that the SA proteins could shuttle between the nucleus and the cytoplasm. We showed that the SA2 protein, but not SA1, can indeed be exported from the nucleus using a Crm1-dependent route. Experiments in HeLa cells were performed to validate the yeast data. They confirmed the functionality of the NLS signal identified in SA1, but the import and putative export of SA2 turned out to be more complex than in yeast.

Since the Crm1-dependent export pathway is conserved in humans we propose that nuclear export can also be used to regulate the activity of SA2 protein in human cells. This is the first report of the possibility of nucleocytoplasmic shuttling of a member of the cohesin complex.

## Results

### Expression of human SA1 and SA2 proteins in yeast

Four variants of nucleotide sequences encoding human SA1 and five variants of SA2 are registered in UniProt and/or deposited in other databases. These DNA or cDNA sequences derive from various tissues and come from large-scale sequencing or dedicated studies (http://www.uniprot.org/uniprot/Q8N3U4#section_comments). These sequences differ in the 5′ UTR and also have single amino acid replacements and alternative in-frame exons. Here we investigated one well described and verified SA1 variant and two variants of SA2 [19], [2], [5], [20]. The two SA2 proteins differ only by length, the shorter one lacking a 69-amino acid N-terminal stretch. Further we will refer to the longer SA2 as SA2L and to the shorter one as SA2S. The SA1 and SA2L proteins are very similar in length (1258 and 1231 aa) and in amino acid sequences, except for the N-terminal tails of 70 and 68 amino acids and stretches of *ca.* 240 and 220 amino acids near the C-termini in SA1 and SA2, respectively (73% identity, excluding the divergent N-termini).

**Figure 1:**
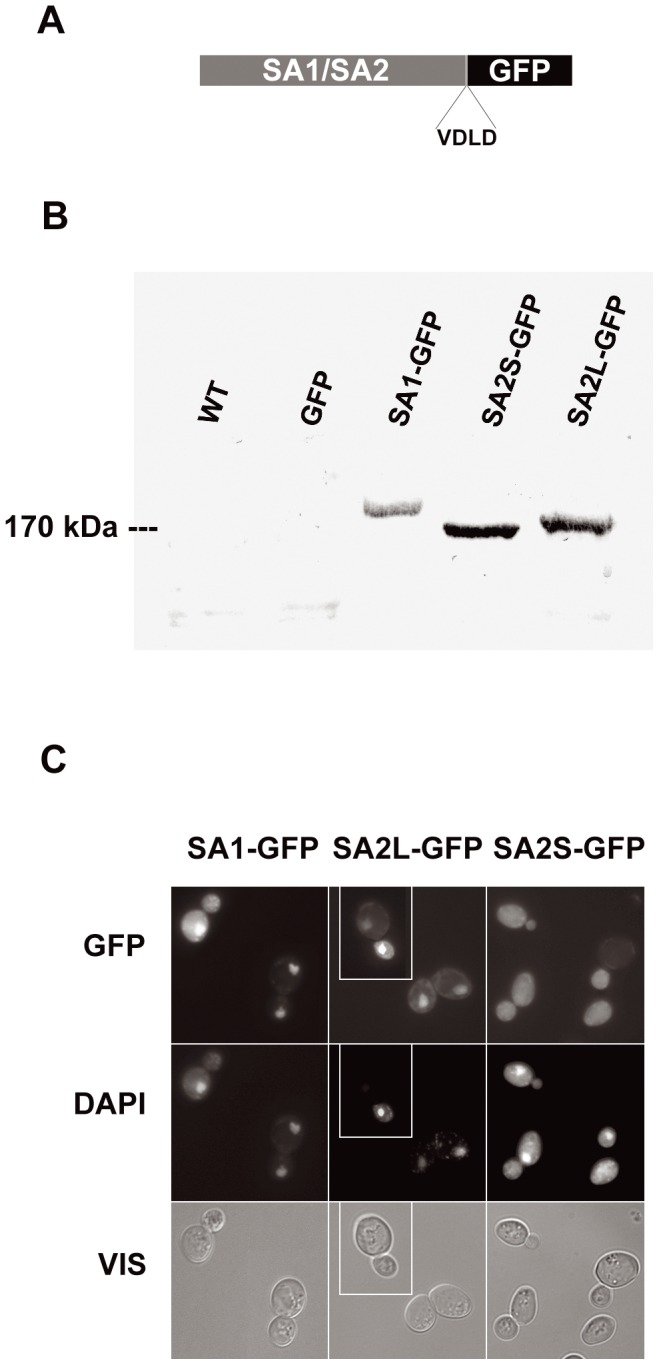
Human proteins SA1 and SA2L expressed in *S. cerevisiae* differ in subcellular localization. (A) Schematic representation of GFP-fused SA proteins. cDNA from *Homo sapiens* encoding SA1, SA2L or SA2S was 3′-fused to GFP-encoding sequence, generating VDLD linker. (B) All SA-GFP proteins have predicted molecular weights. Diploid yeast strain *irr1Δ*/*IRR1* (lacking one copy of Irr1-encoding gene) was transformed with centromeric plasmid pUG35 bearing hybrid genes encoding proteins shown in (A). Yeast were grown to mid-exponential phase in selective medium. Immunoblots of whole-cell extracts were probed with anti-GFP antibodies. Aliquots of 100 µg of protein/lane were resolved by SDS/8% PAGE. Lane WT: yeast strain without a plasmid, lane *GFP*: same strain bearing pUG35, lane *SA1-GFP*: the same strain with plasmid bearing *SA1-GFP*, lane *SA2S-GFP*: as before but plasmid bearing *SA2S-GFP*, lane: *SA2L-GFP*: as before but plasmid bearing *SA2L-GFP*. (C) Microscopic images of yeast cells expressing SA1-GFP, SA2L-GFP or SA2S-GFP fusion proteins. DNA was stained with DAPI, GFP represents fluorescence of fusion proteins, VIS – transmitted light. The middle panel is a composite of two fields from a single experiment but photographed as separate images, as marked.

To check the functioning of the human SA proteins in yeast cells, we constructed plasmids bearing *SA1*-*GFP*, *SA2S*-*GFP* and *SA2L-GFP* hybrid genes. All genes were constructed in the same manner without introducing any additional linear motifs to the fusion proteins (Figure 1A). Plasmids were introduced into the hemizygous yeast strain *irr1Δ*/*IRR1* described in detail by Cena et al. [21]. This strain was chosen for expression because it is devoid of one copy of the *IRR1* gene and thus can be used by a simple sporulation test to check whether the SA cDNA can substitute for *IRR1*, which is essential in yeast. The original aim of our project was to take advantage of the expected functional replacement of the yeast Irr1 protein by its human homologue(s) and to study the functioning of individual SA proteins and their variants in the simple yeast cell. Unfortunately, none of the three SA variants could substitute for the yeast protein in haploid cells (data not shown). Nevertheless, the human proteins were expressed intact in the diploid yeast cell (Figure 1B), permitting the study of their subcellular localization. As expected, SA1 and SA2L were predominantly accumulated in the nucleus. In contrast, the short SA2S variant was distributed throughout the cell (Figure 1C). We assumed that the unexpected localization of SA2S could be due to it lacking a nuclear localization signal located in the missing 69-amino acid N-terminal stretch.

**Figure 2:**
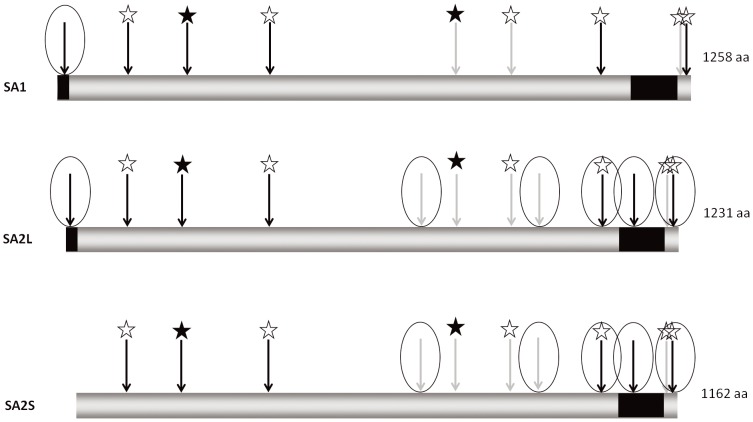
Predicted signals for nuclear localization and Crm1p-dependent nuclear export in SA proteins. Black arrows – NLS, grey arrows – NES. Black asterisks – identical signals, white asterisks – homologous signals. Encircled are signals analysed in this study. Black bars indicate non-conserved regions.

To identify sequences that could serve as NLS in the SA proteins and to check whether the investigated SA proteins bear other localization motifs we searched their amino acid sequences using The Eukaryotic Linear Motif Resource (ELM) (http://elm.eu.org) [22], PSORTII (http://psort.ims.u-tokyo.ac.jp/) [23] and NetNES1.1 (http://www.cbs.dtu.dk/services/NetNES/) [24] servers, with additional manual verification. We found that the SA1 and SA2S proteins contain six putative nuclear localization signals (NLS) each and SA2L – seven. One signal is common to SA1 and SA2, four are located in homologous positions and have similar sequences, one near the C-terminus is unique to SA2, and in their non-conserved N-terminal tails SA1 and SA2L have one NLS each. In this respect SA2S differs from SA2L only in missing the N-terminal-most NLS. In addition to the NLS, we also found putative Crm1-dependent nuclear export signals (NES) – three in SA1 and five in SA2. The three NES of SA1 have fully (one) or highly (two) conserved counterparts in SA2. These data are summarized in Figure 2.

### Nuclear localization signals of SA proteins

**Figure 3:**
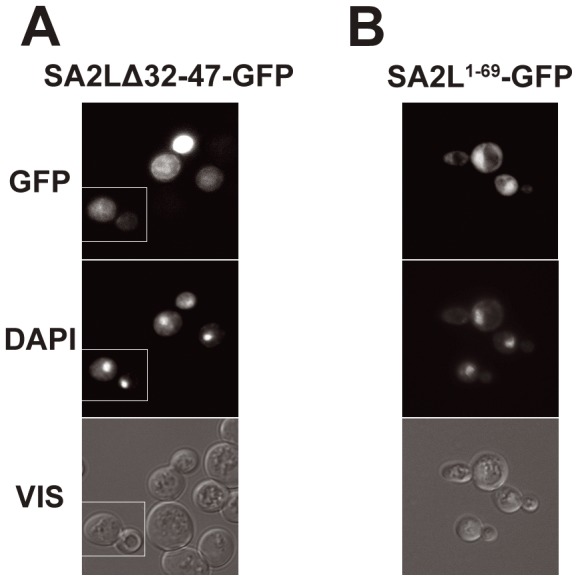
SA2L contains NLS functional in yeast between 32K and 47K. (A) – cells expressing fusion protein SA2LΔ32–47-GFP. (B) – cells expressing SA2^1–69^-GFP. Compare Figure 1C. DNA was stained with DAPI. GFP represents fluorescence of fusion proteins, VIS – transmitted light. Column (A) shows a composite of two fields from a single experiment but photographed as separate images, as marked.

The N-terminal part of SA2L contains a stretch of basic amino acids 32KNQKQGKGKTCKKGKK47 which may represent a mono- or a bipartite NLS. To check whether this sequence is important for nuclear localization of SA2L, we first deleted amino acids 40KTCKKGKK47. However, the protein SA2LΔ40–47 (devoid of this sequence) still localized to the nucleus.We then expanded the deletion by eight amino acids towards the N-terminus and removed 32KNQKQGKGKTCKKGKK47. This sequence resembles a classical bipartite NLS, although it does not fully match the consensus and in fact was only identified by manual inspection; the programs used indicated only the putative NLS between positions 40 and 47. The SA2LΔ32–47 protein localized to the cytoplasm, indicating that, contrary to the *in silico* prediction, the larger bipartite NLS seems to be functional rather than the short monopartite one. In a reciprocal approach, we asked whether the 69 N-terminal amino acids of SA2L can target GFP reporter to the nucleus. The fusion protein SA2L^1–69^-GFP localized to the nucleus, which confirmed that the analyzed fragment contains a functional autonomous NLS (Figure 3).

**Figure 4:**
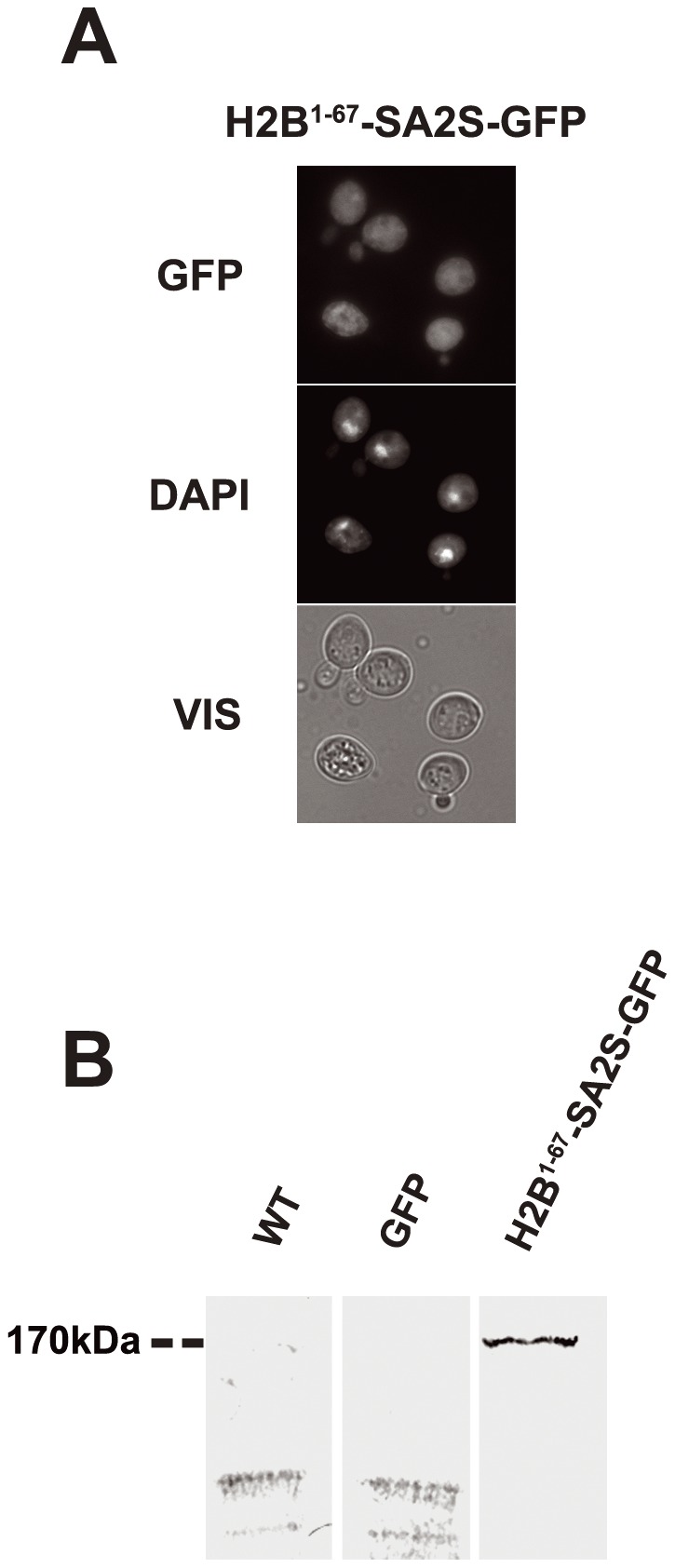
NLS of H2B does not confer nuclear localization on SA2S. (A) Yeast cells expressing fusion protein H2B^1–62^-SA2S-GFP. (B) H2B^1–62^-SA2S-GFP protein has predicted molecular weight. Diploid yeast strain *irr1Δ*/*IRR1* (lacking one copy of *IRR1* gene) was transformed with centromeric plasmid pUG35 bearing hybrid gene encoding the fusion protein. Details as in Figure 1.

To check whether any functional NLS would be sufficient to address SA2 to the nucleus, the short form SA2S (localizing throughout the yeast cell) was fused to a 67-amino acid-long fragment containing a well-characterized NLS of *S. cerevisiae* histone H2B [25]. Amino acids 22KKTSTSTDGKK33 of H2B had earlier been shown to direct a reporter protein to the nucleus [26], [27]. The length of the H2B fragment fused to SA2S corresponded almost exactly to the length of the N-terminus of SA2L which was missing in SA2S. However, although we found that the fusion protein H2B^1–67^-SA2S-GFP was expressed at its expected size, it did not localize to the nucleus (Figure 4). This suggests that the import of SA2 to the nucleus may require a specific NLS, such as the N-terminal one present in SA2L.

**Figure 5:**
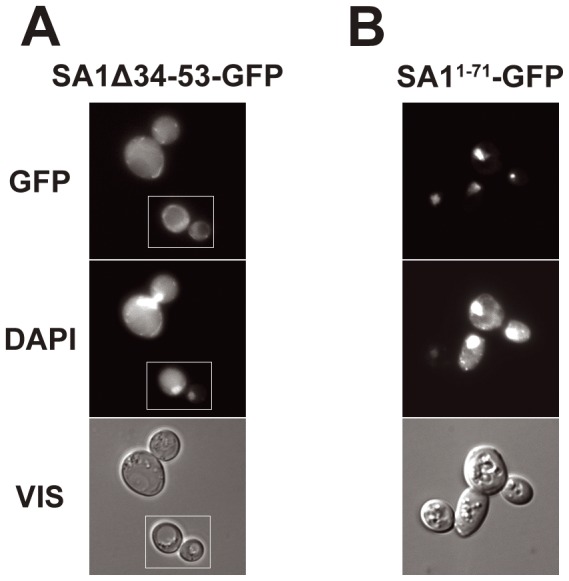
SA1 contains NLS functional in yeast between 34K and 53K. (A) – Cells expressing SA1Δ34–53-GFP. (B) – Cells expressing fusion protein SA1^1–71^-GFP. DNA was stained with DAPI, GFP represents fluorescence of fusion proteins, VIS – transmitted light. Column (A) shows a composite of two fields from a single experiment but photographed as separate images, as marked. For subcellular localization of intact.

Since the SA1 protein was also nuclear in yeast, we asked whether the putative 19-amino acid-long bipartite NLS present in the N-terminal non-conserved part of this protein was responsible. The sequence 34KRKRGRPGRPPSTNKKPRK53 is specific to SA1 and is not homologous to the identified functional NLS32–47 of SA2L. Deleting K34–K53 resulted in the localization of SA1Δ34–53 in the whole cell, which confirmed that the signal is necessary for SA1 nuclear import. When we fused 71 N-terminal amino acids of SA1 containing this signal to GFP, the fusion protein SA1^1–71^-GFP localized to the nucleus, confirming that it contained a functional NLS (Figure 5).

Thus, although the presence of the SA2S protein in the cytoplasm could simply be due to it never being imported to the nucleus because of the lack of the N-terminal NLS that is present in SA2L, we also considered the possibility of this protein actually shuttling between the nucleus and the cytoplasm due to the co-existence of its other putative NLS motifs and NES motifs.

### Functional analysis of putative NES in SA proteins

**Figure 6:**
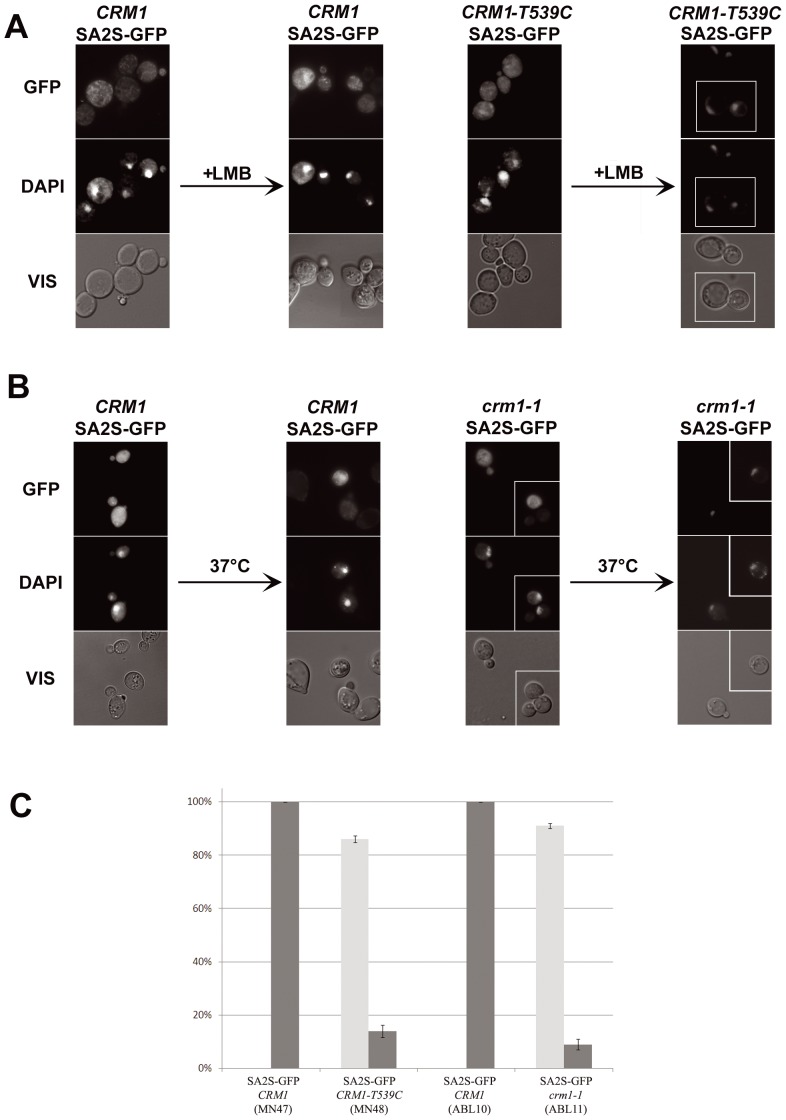
SA2S shuttles between nucleus and cytoplasm in yeast cells. (A) Subcellular localization of SA2S-GFP was analyzed after addition of LMB (Crm1p inhibitor) to 40 ng/ml to cells in logarithmic phase of growth. Strain *CRM1-T539C* bears LMB-sensitive version of Crm1p. Fourth column shows a composite of two fields from a single experiment but photographed as separate images, as marked. (B) Localization of SA2S-GFP protein was analyzed in thermo-sensitive *crm1-1* mutant. Transfer of cells grown at 30°C to 37°C for 30 minutes caused nuclear shift of the fusion protein in 100% of cells. Third and fourth columns show a composite of two fields from a single experiment, as marked. On the right in (A) and (B) control experiments in wild-type yeast are shown. DNA was stained with DAPI, GFP represents fluorescence of fusion proteins, VIS – transmitted light. (C) Frequencies of cells localized predominantly to the cytoplasm (black) or to the nucleus (gray) in strains bearing *CRM1-T539C* (LMB-sensitive) or *crm1-1* (thermo-sensitive) versions of Crm1p, following LMB treatment or temperature shift, respectively. MN47 and ABL10 are corresponding control strains bearing wild type *CRM1* gene, subjected to the same treatments.

The presence of numerous putative NLS and NES signals suggested that the actual intracellular localization of the SA proteins could be determined by the balance between the two types of localization signals. To check whether the putative NES were functional we used two approaches, both basing on the fact that the NES identified were of the Crm1-dependent kind. Crm1 is an exportin well conserved between yeast and humans [28]. First, we treated yeast cells expressing SA1 or SA2 variants devoid of their respective N-terminal NLS, characterized above, with leptomycin B (LMB), an inhibitor of certain Crm1 variants [29]. Since Crm1 of standard laboratory yeast strains is insensitive to LMB, we used for these experiments strain *CRM1-T539C* (MNY8) which bears a leptomycin B-sensitive version of Crm1 [30]. To quantitate the nuclear/cytoplasmic localization of SA1Δ34–53 and SA2S fused with GFP in a population of cells, at least 100 cells were scored according to their fluorescence localization as predominantly cytoplasmic or predominantly nuclear. We found that the addition of LMB to a final concentration of 100 ng/ml to cells in logarithmic phase of growth caused a clear-cut shift of GFP fluorescence to the nucleus in 86% of cells expressing SA2S-GFP (Figure 6A, right), but it did not affect the cytoplasmic localization of SA1Δ34-53-GFP (not shown). As expected, LMB had no effect on localization of SA2S-GFP in cells expressing wild type Crm1 protein insensitive to LMB (Figure 6A, left). To confirm the Crm1-dependent export of SA2S we used another approach. The plasmid encoding SA2S-GFP was introduced into yeast strain ABL11 bearing a thermo-sensitive *crm1-1* allele [31]. Transferring of these cells from 30°C to 37°C caused a nuclear shift of the fusion protein in 91% of the cells of an unsynchronized culture (Figure 6B, right). As expected, a shift from 30°C to 37°C failed to affect the nuclear localization of SA2S-GFP in cells expressing the temperature-insensitive wild type Crm1 protein (Figure 6B, left). Figure 6C summarizes those results.

The above results indicated that: **a**. the SA2 protein devoid of the first NLS (SA2S) loses its nuclear localization because the protein is efficiently exported from the nucleus in a Crm1-dependent manner, which logically requires that **b**. a functional NLS must still be present in SA2S; and **c**. the SA1 protein deprived of its first NLS is no longer capable of entering the nucleus.

When SA2LΔ32-47-GFP was studied, the results were qualitatively similar to those obtained for SA2S-GFP, albeit less clear-cut (not shown). First, LMB caused the GFP signal to become predominantly nuclear in only 32% of cells, suggesting additional Crm1-independent nuclear export by an unidentified NES. Second, the ABL11 cells expressing SA2LΔ32–47-GFP became extremely temperature-sensitive and showed increased cell membrane/cell wall fragility when shifted to a higher temperature, resulting in a 20% loss of viability. Nevertheless, nuclear localization of the GFP signal was also observed in 29% of such cells.

When the putative NES of SA1 and SA2 are compared, one notices that in addition to signals common to these two proteins, SA2 contains two signals that are not conserved in SA1. We reasoned that the NES which are found in both SA2 and SA1 are likely to be non-functional since SA1 seems not to be exported. We therefore focused our attention on the two NES unique to SA2.

The two SA2-specific NES (see Figure 2) comprise sequences numbered 689LLRLKKQMRV699 and 953LEKFMTFQMSL964 in SA2S. The numbering in SA2L is 758–768 and 1022–1033, respectively. These sequences constitute putative signals for Crm1p-dependent export, the consensus of which is ΦX_2–3_ΦX_2–3_ΦXΦ, where Φ represents L, I, V, F or M and X – any amino acid [32]. The corresponding sequences in SA1 deviate from the consensus. Below we show an alignment of the first of the SA2-specific NES with the corresponding sequence from SA1. To verify the functionality of the predicted NES we first disrupted the original SA2S sequence 689LLRLKKQMRV699 by substituting V699 with S (both underlined), in this manner mirroring the (predicted to be non-functional) SA1 sequence.

**ΦX_2–3_ΦX_2–3_ΦXΦ consensus**

SA2S LLR LKKQMRV**689–699**

SA1 LLV LRKTVKS**762–771**

**Figure 7:**
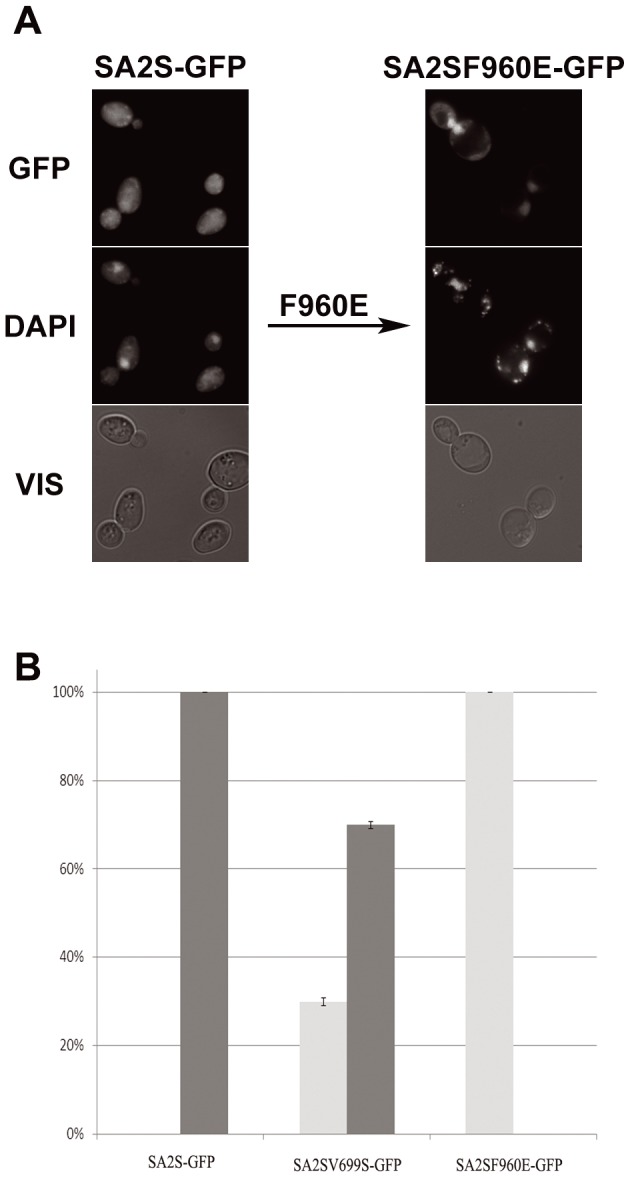
SA2S contains NES functional in yeast between L953 and M962. Consensus for Crm1p-dependent export: ΦX_2–3_ΦX_2–3_ΦXΦ, where Φ represents L, I, V, F or M and X – any amino acid. (A) Left – cells expressing fusion protein SA2S-GFP. Right – cells expressing SA2SF960E–GFP protein bearing the substitution F960E which disrupts NES 953 LEK FMT FQM 962. The SA2SF960E–GFP protein accumulates in the nucleus in 100% of cells. DNA was stained with DAPI, GFP represents fluorescence of fusion proteins, VIS – transmitted light. (B) SA2S-GFP protein with NES signals disrupted by site-directed mutagenesis. SA2V699S – inactivated NES between positions 689 and 699, SA2SF960E – between positions 953–964. At least 100 cells were counted. Bars show percentage of cells with a given localization of GFP signal. Protein localized to the cytoplasm – black, to the nucleus–grey.

We found that the substitution V699S in SA2S shifted the GFP fluorescence to the nucleus in 30% of cells (micrographs not shown, but data included in Figure 7B). This experiment apparently confirmed the functionality of the original NES signal. However, we found that the plasmid expressing the mutated V699S SA2S protein also carried a spontaneous single mutation C450G leading to the substitution S150R. This region of SA2S does not carry recognizable NLS or NES-like motifs. Despite various experimental approaches taken we were unable to propagate in bacteria a plasmid encoding the protein with the V699S substitution alone. Thus, we cannot exclude the possibility that the substitution S150R could also have affected the SA2S localization.

In the second region of interest two putative nested signals for Crm1-NES are predicted. The shorter one is:

**ΦX_2–3_ΦX_2–3_ΦXΦ**

SA2S LEK FMT FQM **953–962**

and the longer one:

**ΦX_2–3_ΦX_2–3_ΦXΦ**

SA2S LEKFMTFQMSL **953–964**

In the shorter track we substituted F960 (underlined) in SA2S with E. The rationale for this substitution was the same as it was with the first signal – in SA1 glutamic acid is present in the corresponding position. The SA2S-F960E-GFP localized to the nucleus in 100% of cells, confirming functionality of this NES (Figure 7).

Taken together, the above results suggested that both SA2-specific NES are required for an effective export, albeit the former one (689–699) seems to be less important.

### Verification of functionality of SA NLS and NES in HeLa cells

**Figure 8:**
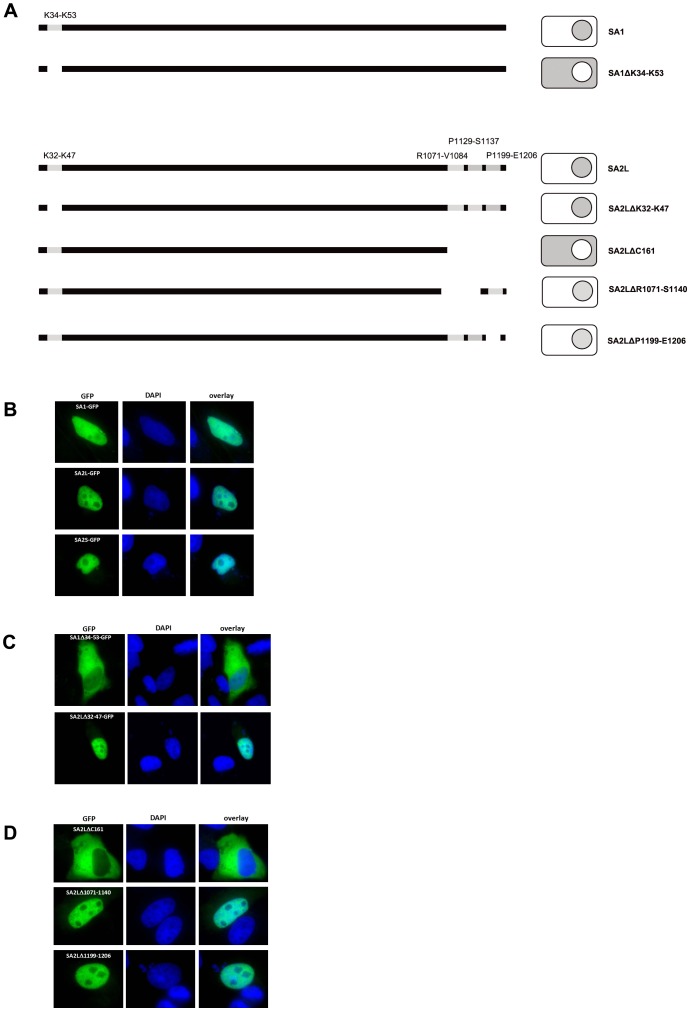
NLS of SA1 identified in yeast is the same in HeLa cells but SA2 is targeted to the nucleus of HeLa by C-terminally localized NLS. (A) Schematic representation of HeLa cells expressing SA1-GFP and SA2-GFP and their deletion mutants. Arrows indicate localization of NLS discussed in the text. Grey color indicates GFP fluorescence. (B) HeLa cells expressing SA1-, SA2L- and SA2S-GFP fusion proteins. (C) HeLa cells expressing SA1-GFP and SA2L-GFP devoid of N-terminal NLS 34–53 and 32–47, respectively. (D) HeLa cells expressing SA2L protein devoid of 161 C-terminal amino acids (upper panel), C-terminal NLS 1071–1140 (middle panel), C-terminal NLS 1199–1206 (lower panel). GFP represents fluorescence of fusion proteins, DNA was stained with DAPI.

To check if the findings derived from yeast also hold true in mammalian cells we used HeLa cells transiently transfected with plasmids bearing *SA1*-*GFP*, *SA2S*-*GFP* or *SA2L-GFP* hybrid genes, and their deletion mutants devoid of sequences encoding putative NLS signals. Figure 8A depics all constructs which will be discussed below.

**Table 1:** Plasmids used in this study.

Plasmid	Relevant plasmid genotype	Reference
pUChSA-1	pUC19-*SA1*cDNA	J-L. Barbero, Centro de Investigaciones Biologicas (CSIC), Madrid, Spain
pUChSA-2	pUC18-*SA2*cDNA	as above
pUG35	P*_MET25_*- MCS-*GFP*-T*_CYC1_ URA3* CEN	J. H. Hegemann, Heinrich-Heine-Universitat, Dusseldorf, Germany
pIGout_A_	P*_GAL1_*-1-67NLS*_H2B_*-2x*GFP URA3* 2 µ	based on pRS426(Clontech)
pPK1	P*_MET25_*–*SA1*–*GFP*–T*_CYC1_ URA3* CEN	This study, based on pUG35
pLT1	P*_MET25_*–*SA2*–*GFP*–T*_CYC1_ URA3* CEN	This study, based on pUG35
pLT1-M1	P*_MET25_*–*SA2V698S*–*GFP*–T*_CYC1_ URA3* CEN	This study
pLT1-M2	P*_MET25_*-*SA2F959E*–*GFP*–T*_CYC1_ URA3* CEN	This study
pLT3	P*_MET25_*–1-67NLS*_H2B_*–*STAG2*–*GFP*–T*_CYC1_ URA3* CEN	This study
pPK2	P*_MET25_*–NLS*_SA1_*–*GFP*–T*_CYC1_ URA3* CEN	This study
pSA1	P*_CMV_*-*SA1*-*GFP*-SV40 Late poly(A)	based on pCl-neo(Promega)
pSA2L	P*_CMV_*-*SA2L*-*GFP*-SV40 Late poly(A)	as above
pSA2S	P*_CMV_*-*SA2S*-*GFP*-SV40 Late poly(A)	as above
pSA1Δ34–53	P*_CMV_*-*SA1ΔK34*-*53K*-*GFP*-SV40 Late poly(A)	as above
pSA2LΔ32–47	P*_CMV_*-*SA2ΔK32*-47*K*-*GFP*-SV40 Late poly(A)	as above
pSA2LΔC161	P*_CMV_*-*SA2ΔC161*-*GFP*-SV40 Late poly(A)	as above
pSA2LΔ1071–1140	P*_CMV_*-*SA2ΔR1071-S1140-GFP*-SV40 Late poly(A)	as above
pSA2Δ1199–1206	P*_CMV_*- *SA2ΔP1199-E1206 -GFP*-SV40 Late poly(A)	as above

Abbreviations for description of plasmids: CEN, centromeric; 2 µ, episomal; MCS, multiple cloning site.

All three GFP fusion proteins with wild type SA had nuclear localization (Figure 8 B). This result was different from that obtained in yeast where SA2S-GFP was distributed throughout the cell. Subsequently, we introduced a plasmid encoding SA1Δ34–53 protein (devoid of the N-terminal NLS) and found that the protein was distributed througout the cell, predominantly in the cytoplasm (Figure 8C, upper panel), which confirmed that the same signal is necessary for the nuclear import of SA1 both in *S. cerevisiae* and in HeLa cells.

The lack of a difference of the cellular localization between SA2L and SA2S indicated that the N-terminal NLS localized between K32 and K47 of SA2L is not necessary in human cells, although it was both necessary and sufficient in yeast. The unaffected nuclear localization of the SA2LΔ32–47 protein, devoid of this N-terminal signal (Figure 8C, lower panel), confirmed that supposition and indicated that another NLS directs SA2 to the nucleus of HeLa cells. Since in SA1 the only functional NLS was the N-terminal one, the SA2 signals shared with SA1 were unlikely to be functional. We therefore focused on three NLS localized in the C-terminal part of SA2 since one of them (P1129-S1137, numbering for SA2L) was unique to SA2 and two others (R1071-V1084 and P1199-E1206) were similar to but not identical in the amino acid sequences with the respective signals present in SA1. Deletion of the C-terminal 161 amino acids of SA2, comprising all three NLS, resulted in the protein named SA2LΔC161. The truncated protein had exclusively cytoplasmic localization (Figure 8D, upper panel) indicating that the NLS necessary for the import of SA2 to the nucleus (one of the three putative ones or their combination) is localized at the C-terminus. To pinpoint the C-terminal NLS actually responsible for nuclear localization of SA2 we first deleted amino acids R1071 through S1140, encomprising two of the three C-terminal NLS. This protein still localized to the nucleus, indicating that the C-terminal-most signal (P1199-E1206 in intact SA2L) is sufficient for nuclear addressing. In a reciprocal experiment, we deleted amino acids 1199–1206 encompassing only that NLS. Unexpectedly, that protein was also present in the nucleus. This indicates that the nuclear localization of SA2L is executed by redundant signalings in the C-terminal part, of which the first and/or second, or the third, suffice for the addressing. Our attempt to differenciate the role of signals R1071-V1084 and P1129-S1137 (first and second, respectively) failed since the deletion of the latter NLS produced an apparently unstable protein and despite several attempts such a protein could not be detected.

Subsequently, we attempted to verify whether the SA2LΔC161 protein is extranuclear because it never gets imported to the nucleus or because its export from the nucleus predominants over nuclear import weakened by the absence of the C-terminal import signals. The NES signal L1022-L1033, which was functional in yeast, is intact in SA2LΔC161. However, treatment of cells expressing SA2LΔC161-GFP with LMB did not cause a shift of the GFP fluorescence to the nucleus (not shown). Modifications of the original LMB protocol for mammalian cells [33] by increasing the dose of LMB or duration of the treatment did not change the results. This indicated that SA2L protein devoid of the C-terminal part is not imported to the nucleus.

After showing that the redundant NLS functional in HeLa cells are located in the C-terminal part of SA2, we returned to the yeast system. We checked whether the protein SA2SΔC161 (short form, devoid of the N-terminal NLS and lacking the C-terminus) can shuttle between the nucleus and the cytoplasm by performing experiments with leptomycin B. The SA2 variant was, as expected, distributed throughout the cell without LMB treatment and that localization was not altered upon blocking of Crm1-dependent export with LMB (results not shown). This behaviour indicates that the second, weaker NLS functional in yeast that was responsible for the nuclear localization of SA2S in the presence of LMB (see “Functional analysis of putative NES in SA proteins” above) was located in the C-terminal 161 amino acids. Thus, the NLS that were functional in HeLa cells were also recognized as secondary NLS in yeast.

## Discussion

Protein shuttling between the nucleus and the cytoplasm is controlled by nuclear localization (NLS) and nuclear export sequences (NES) that bind directly, or through adaptor proteins, to specific karyopherins that allow selective and directional passage through the nuclear pore complex (reviewed in [34]–[36]). The presence of both types of “address tags” in a single protein allows modulation of its subcellular localization by largely unstudied mechanisms. Such mechanisms, however, are fundamental to cell functioning since the potential interactors of any given protein are different in the nucleus and cytoplasm, and many proteins shuttle between the two compartments in a cell-cycle- or signal-dependent manner.

*S. cerevisiae* can be used as a representative model to study the nucleus/cytosol exchange because the nuclear transport pathways are very highly conserved among lower and higher eukaryotes [37], [38]. Of the fourteen karyopherin family members identified in *S. cerevisiae*, ten have human homologs [39]. Examples of proteins with multiple NLSs have been described, some of which are served by different importin-β (karyopherins that facilitate the import of proteins into the nucleus) family members [40], for review: [41], but in general, little is known about how each individual signal is used. Here we identified *in silico* numerous putative NLS and NES sequences in human SA proteins. We confirmed that the N-terminal-most NLS of SA1 and SA2L, although unlike each other, are recognized by the yeast nuclear import machinery and are necessary to direct these proteins to the nucleus and sufficient to confer nuclear localization on GFP. Affinity capture and mass spectrometry analysis has identified karyopherin Kap123 as an interactor of Irr1, the yeast homologue of SA [42]. It is likely that this karyopherin was also responsible for the nuclear localization of SA1 and SA2.

Since deletion of the N-terminal NLS caused retention of the majority of the SA1 protein in the cytoplasm – the functionality of the other NLS predicted in this protein remains unclear. On the other hand, the SA2S protein which does not contain the N-terminal stretch of amino acids, apparently required for addressing its longer variant SA2L to the nucleus, and also SA2LΔ32–47, can still accumulate in the nucleus when their Crm1p-dependent export from the nucleus is prevented by leptomycin B or a temperature shift of the temperature-sensitive *crm1-1* strain. This indicates that other NLS signals, besides the N-terminal one, may also be functional in SA2. By comparison with the SA1 protein, in which apparently only the N-terminal NLS is functional, the most likely candidate for the additional functional NLS in SA2 is the penultimate one, since it is the only NLS unique to SA2 apart from the N-terminal one. However, when we deleted this penultimate NLS in SA2S, the mutant protein could still enter the nucleus upon LMB treatment or upon temperature shift of the *crm1-1* strain (results not shown). This result shows that, contrary to expectations, some other putative NLS must be functional in SA2.

By expressing the truncated SA2S variant we also found evidence for a previously unrecognized Crm1p-dependent export of SA2 cohesin from the nucleus. Human cohesins are believed to be localized to the nucleus until the G2/M transition, when the nuclear envelope is disassembled. The envelope re-forms in the telophase, which is coincident with the reassociation of cohesin with chromosomes. No data indicating cohesin shuttling between nucleus and cytoplasm have been published until now. The closed mitosis of *S. cerevisiae* used in this study facilitated the observation of the possibility of the SA2 protein moving between the nucleus and the cytoplasm. Since *S. cerevisiae* cells have many elements of the mitotic division common with mammalian cells, conclusions drawn from yeast research can be applied to higher eukaryotes to elucidate the functioning of mammalian proteins (for review: [43], [44]). Therefore, the indication that SA2 can be exported from the yeast nucleus by a Crm1-dependent mechanism makes it plausible that a similar mechanism functions in mammalian cells (where a Crm1p orthologue is known to function) [31]. Such nucleocytoplasmic shuttling of cohesins could thus be a previously unrecognized means of their regulation.

It is also important to notice that human SA2 and its yeast homologue Irr1 have a role in the regulation of transcription [14], [15], [45], [46]. Thus, it is possible that the nucleocytoplasmic shuttling of SA2 may serve to regulate that fraction of this protein which is involved in the regulation of transcription, in a manner similar to that long known for numerous bona fide transcription factors (see [47]–[49] for review).

Our experiments in HeLa cells confirmed that the main NLS of SA1 cohesin required and sufficient for its nuclear import is indeed localized at the N-terminus and is the same as the one identified in yeast. However, the N-terminal NLS of SA2L crucial in yeast seems not to function in human cells. Instead, our experiments evidence an essential role of the C-terminus in SA2L trafficking in HeLa cells. This region of 161 amino acids comprises three putative NLS acting redundantly: the presence of either the C-terminal one or the first plus the middle one was required for nuclear import. In the SA2 C-terminus numerous mitosis-specific phosphorylation sites have been identified [50] essential for unloading of cohesin from chromosome arms during early mitosis. Since some of those sites are in close proximity of the three C-terminal NLS, one is tempted to speculate on the possible effects of the phosphorylation on the functioning of the adjacent import signals. Our data predict a very complex mechanism of SA2 nuclear import requiring further detailed studies.

We assume that, similarly to yeast, also in human cells SA2 may be actively exported from the nucleus, although mechanisms that provoke such nucleocytoplasmic shuttling remain unknown. Since during meiotic division a specific SA paralogue, SA3, replaces most SA1 and SA2 molecules [51], one cellular event that could require the SA1 and SA2 proteins to leave the nucleus could be the switch from mitotic to meiotic division. The mechanism of Crm1p-dependent export has been described for another protein involved in chromosome segregation in human cells – separase. The nuclear exclusion of separase has been postulated to prevent cohesin cleavage in interphase cells [52]. Thus, shuttling between the nucleus and the cytoplasm constitutes an important regulatory mechanism for other multifunctional proteins involved in DNA repair and maintenance of genetic stability [53]–[56].

## Materials and Methods

### Strains, growth conditions and genetic procedures

*Escherichia coli* XL1-Blue MRF' (Stratagene, Saint Quentin en Yvelines, France) was used for molecular manipulations. All *S. cerevisiae* strains used in this study were derivatives of W303. Strain *irr1Δ/IRR1* was described in Cena et al. [21]. ALB11, bearing temperature-sensitive Crm1p, and the control strain ALB10 [57] were provided by Dr. Anita Hopper, Ohio State University, Columbus, OH, USA. Strains MNY8 (*CRM1-T539C*), bearing leptomycin B-sensitive version of Crm1p, and the control MNY7(*CRM1*) [30] were provided by Dr. Michael Rosbash, Brandeis University, Waltham, MA, USA. Yeast growth and transformation followed standard procedures [25]. To study the effects of Crm1p on localization of human SA proteins, plasmids (described further) bearing cDNA encoding SA1 protein or variants of SA2 fused to the N-terminus of GFP were transformed into *crm1-1/xpo1-1* (ABL11) and *CRM1/XPO1* (ABL10) yeast. The *crm1-1*/*xpo1-1* allele supports ABL11 growth at room temperature but not at 37°C [31]. Strains were grown overnight to log phase at 30°C in selective minimal medium. Cultures were divided into halves and incubated at 37°C for 30 min or allowed to continue growing at 30°C. After 30 min cells were fixed with 4% (w/v) formaldehyde and subjected to fluorescence microscopy. For leptomycin B (LMB) treatment the same plasmids were transformed into *CRM1-T539C* and *CRM1* strains. As above, strains were grown overnight, cultures were divided and treated with LMB (LC Laboratories, Woburn, MA, USA, cat. No. L-6100) at 40 ng per ml of medium. Sixty minutes after LMB treatment, cells were collected, fixed with 4% formaldehyde and subjected to fluorescence microscopy.

### HeLa cell culture

HeLa cells (European Cell Culture Collection, catalogue no. 93021013) were grown in Dulbecco's modified Eagle's medium (DMEM) supplemented with 10% fetal bovine serum (FBS). Cells were incubated in polystyrene flasks (Sarstedt) in 5% CO_2_-balanced air at 37°C. All cell culture reagents were from Gibco/Life Technologies.

### Transient cell transfection

After reaching 100% confluence, cells were seeded on collagen-coated glass coverslips (placed in standard six-well plates) and grown in culture medium for 20–30 hours until 40–60% confluence. Cells were then transfected using the FuGENE reagent (Roche Diagnostics) according to manufacturer's instructions. For maximal transfection rates, FuGENE was mixed with plasmid DNA at a 7∶2 (µg∶µl) ratio. In some experiments, leptomycin B was added to the medium (final concentration of 6 ng per ml, unless stated otherwise). The effect of LMB was studied at different intervals (from 20 minutes to 18 hours). At 24 hours after transfection, cells were fixed in 4% paraformaldehyde (Sigma) for 20 minutes at room temperature, permeabilized with 0.1% Triton X-100 (Sigma) and mounted in SlowFade (Invitrogen) and 0.01 mg/ml DAPI (4′, 6′-diamino-2-phenylindole dichloride, Sigma).

### Plasmid construction

Plasmids listed in Table 1 were constructed by standard methods. All PCR products were sequenced after cloning. cDNAs serving as templates for amplification of *SA1* gene (EMBL/GenBank Accession Number Z75330.1, NM_005862) and *SA2* short splice variant (EMBL/GenBank Accession Number Z75331) were obtained from Dr. José-Luis Barbero, Dpto. Biología Celular y del Desarrollo, Centro de Investigaciones Biologicas (CSIC), Madrid, Spain. *SA2* variant 4 (EMBL/GenBank Accession Number NM_006603.3 or NM_006603.4) was purchased from OriGene. To create *SA1* and *SA2* fusions to *GFP*, PCR-generated fragments were inserted into the centromeric plasmid pUG35 (kindly provided by Dr. J. H. Hegemann, Heinrich-Heine-Universitaat, Dusseldorf, Germany) bearing *S. cerevisiae MET25* promoter and a GFP-encoding sequence. To create SA2 fusion to nuclear localization sequence (NLS) of *S. cerevisiae* histone H2B (codons 1–67) the NLS-encoding fragment was PCR-amplified using pIGout_A_ plasmid [56] as a template. To fuse SA1 and SA2 fragments containing putative NLS with GFP the 5′-terminal 213-bp fragment of *SA1* or the 207-bp fragment of *SA2L* was PCR-amplified, introduced into pUG35 and verified by sequencing. In general, plasmids bearing *SA* genes propagate poorly in *E. coli*. We also noticed spontaneous point mutations occurring in *SA* genes irrespective of the bacterial strain used for propagation. Thus, careful DNA sequencing was done after each manipulation.

### Site-directed mutagenesis

Single mutations causing substitutions V698S and F959E of SA2 were generated by overlap extension PCR [58] with pairs of internal complementary oligonucleotides for the desired mutation (F-forward and R-reverse) and two external (E) oligonucleotides (Table S1). The *SA2* cDNA in pUC19 was used as the template in the first round of PCR. Both mutant alleles were fully sequenced to verify the presence of desired mutation.

### Western blotting

To visualize chimeric GFP-tagged proteins on Western blots, protein samples (100 µg/lane) were subjected to 8% SDS-PAGE. Electrophoresis was followed by blotting onto Hybond-C extra membrane, probing with an anti-GFP antibody (A.v. Peptide antibody Living Colors AB, Becton Dickinson), anti-rabbit alkaline phosphatase-conjugated secondary antibody (Promega) and development with CDP-Star (Roche) or Western Blue Stabilized Substrate for Alkaline Phosphatase (Promega).

### Fluorescence microscopy

Yeast cells were observed and images were taken using a Nikon Eclipse E800 fluorescence microscope with a 100× objective. GFP-fusion proteins were visualized in liquid-grown cells fixed with 4% formaldehyde for 20 min. DAPI was used to stain DNA. To estimate the percentage of yeast cells with a given SA-GFP localization, at least 100 cells were analyzed. For transfected HeLa cells an IX71 Olympus fluorescence microscope was used to analyze the distribution of GFP-fused proteins. For each protein variant tested, at least 100 transfected (GFP-expressing) cells were analyzed in duplicate in two independent transfection experiments.

## Supporting Information

Table S1 Oligonucleotides used in construction of *SA2* mutants by site-directed mutagenesis.
(DOC)
