# Correction: Nuclear Import and Export Signals of Human Cohesins SA1/STAG1 and SA2/STAG2 Expressed in *Saccharomyces cerevisiae*

**DOI:** 10.1371/journal.pone.0112338

**Published:** 2014-10-27

**Authors:** The PLOS ONE Staff

The authors would like to give readers the following update: Due to the mislabeling of a plasmid used for HeLa cell transformation the authors mistakenly claimed that SA2 protein devoid of two putative C-terminal nuclear localization signals found between amino acids 1071 and 1140 had a nuclear localization (Figure 8A, penultimate scheme, and 8D, middle row). In fact, the unpublished results corroborate the data of Kong et al., (2014) indicating that both those C-terminal NLSs are required for the nuclear localization of the C-terminal fragment of SA2 [2]. Other conclusions of the work are unaffected. The authors regret any inconvenience this error may have caused.
