# Supplementary material for: Nuclear Import and Export Signals of Human Cohesins SA1/STAG1 and SA2/STAG2 Expressed in Saccharomyces cerevisiae
Source: PLoS One. 2012 Jun 8;7(6):e38740. doi: 10.1371/journal.pone.0038740 (PMC3371031; doi:10.1371/journal.pone.0038740)
Supplement: Table S1 — Oligonucleotides used in construction of SA2 mutants by site-directed mutagenesis. (DOC) [file pone.0038740.s001.doc]

**Table S1.** Oligonucleotides used in construction of *SA2* mutants by site-directed mutagenesis

| Name | Sequence 5’-3’ | Introduced restriction site |
| --- | --- | --- |
| S1P1E | TGGAAATATATACCACTGGACGATTAG |  |
| S1P2M1R | ATGTTGACATATCTGACAAAA**AGA**TCTCA  TTTGTTTCTTTAAACG | BglII |
| S1P3M1F | CGTTTAAAGAAACAAATGAGA**TCT**TTTTGT  CAGATATGTCAA | BglII |
| S1P4E | TTCATTAAAAAGCTGTTGCAGACTGAG |  |
| S2P1E | ATTCTGTGTGATATTTTGATGATCTTC |  |
| S2P2M2R | CACATCCTCTCTTC**TT**A**AG**GACATCTG**TTC**G  GTCATGAACTTTTC | BspTI |
| S2P3M2F | GAAAAGTTCATGACC**GAA**CAGATGTC**CT**T**AA**GAAGAGAGGATGTG | BspTI |
| S2P4E | GAACTGATTCATCCATAATTGAAGCTG |  |

Mutated nucleotides in boldface; introduced restriction sites are underlined; F- forward, R- reverse, E- external.
